# Monitor gastrointestinal tolerance in children who have switched to an “enteral formula with food‐derived ingredients”: A national, multicenter retrospective chart review (RICIMIX study)

**DOI:** 10.1002/ncp.10812

**Published:** 2021-12-21

**Authors:** Graeme O'Connor, Marie Watson, Martha Van Der Linde, Rita Shergill Bonner, Julia Hopkins, Sharan Saduera

**Affiliations:** ^1^ Department of Dietetics Great Ormond Street Hospital Foundation Trust London United Kingdom; ^2^ Department of Dietetics Sheffield Children's NHS Foundation Trust Sheffield United Kingdom; ^3^ Department of Dietetics Herefordshire and Worcestershire Health and Care NHS Trust Worcester United Kingdom; ^4^ Department of Dietetics Evelina London Children's Hospital London United Kingdom; ^5^ Medical Affairs Nestlé Health Science Gatwick United Kingdom

**Keywords:** blended diet, children, enteral formula with food‐derived ingredients, gastrointestinal intolerance, microbiomes

## Abstract

**Background:**

Enteral tube feeding intolerances, such as diarrhea, are commonly reported in children. In the pediatric population, interest is growing in the use of blended diets for the management of enteral feeding intolerances. Fiber within a blended diet stimulates the growth of beneficial gut bacteria, which in turn produce short‐chain fatty acids, which are utilized as energy substrates for enterocytes. Enteral formula manufacturers have responded to this trend towards “real‐food” blended diets and developed an enteral formula with food‐derived ingredients. The aim of this study was to collect data relating to feed tolerance in children who had switched to an “enteral formula with food‐derived ingredients.”

**Methods:**

A national multicenter retrospective study.

**Results:**

Dietitians collected data from 43 medically unwell children between March 2021 and July 2021. Significant improvements were reported in children who had switched to an “enteral formula with food‐derived ingredients” in retching 17 of 18 children (95%), flatulence 6 of 8 children (85%), loose stools 10 of 11 children (90%), and constipation 10 of 11 children (90%). These improvements in gastrointestinal symptoms were reflected in weight change during the one month period measurements were collected (baseline, 19.5 kg [SD, 9]; 1 month, 20.1 kg [SD, 9]; *P* = 0.002).

**Conclusion:**

We have observed beneficial outcomes in medically complex children who have switched to an “enteral formula with food‐derived ingredients.” Our data should motivate healthcare professionals to implement more research to better evaluate the clinical impact and mechanisms of action of blended diets and enteral formulas with food‐derived ingredients.

## INTRODUCTION

Enteral nutrition (EN) is the preferred route for the nutrition support of patients who are unable to meet their nutrition requirements orally.^1^ Standard enteral formulas are easily quantifiable, convenient, portable, safe, and reasonably cost effective.^2^ Clinical manifestations of enteral feeding intolerances, such as abdominal distension and diarrhea, are some of the complications that can occur in patients.^3^ The frequency of diarrhea in enterally fed patients ranges from 29% to 72%.^4^, ^5^ The management of persistent feed intolerances results in repeated feed withdrawal to allow for gut rest, contributing to malnutrition through a reduction in nutrition intake, a decrease in nutrient absorption, and an increase in nutrient reserve catabolism.^6^

In the pediatric population interest is growing in the use of a blended diet for the management of feeding intolerances. Blended diets are food‐based formulas liquefied to a consistency that will enable passage through a feeding tube. It is perceived to be more natural and better tolerated compared with commercially available standard enteral formulas.^7^ Previous studies have reported positive clinical outcomes with the use of blended diets, including reduced gagging, retching, and vomiting compared with commercially available standard enteral formulas.^7^, ^8^ In 2020, the British Dietetic Association amended its guidelines to enable dietitians in the United Kingdom to support a blended diet for tube‐fed individuals and to encourage an open, multidisciplinary approach to administering blended diets via a feeding tube (British Dietetic Association Policy Statement^9^). Prior to this, there had been a lack of clear professional guidance.

The mechanisms as to why a blended diet is better tolerated than a standard enteral formula is unclear.^10^ However, it stands to reason that “real food” aids normal gut functioning. Furthermore, there is evidence to suggest that fiber within a blended diet promotes the growth of beneficial gut flora bacteria, thereby inhibiting harmful bacteria.^5^ In the large intestine, the microbiota ferment nondigested dietary fiber to produce short‐chain fatty acids, primarily acetic, propionic, and butyric acid, which epithelial cells use as an energy source.^11^ Butyrate is considered the main energy substrate for enterocytes and a stimulator of growth and differentiation.^12^ Moreover, short‐chain fatty acids are crucial to inhibit proinflammatory mediator activities in the intestinal epithelium.^13^

Fiber that includes fructo‐oligosaccharides, galacto‐oligosaccharides, and inulin (also known as prebiotics) were shown in multiple human studies to increase the concentrations of bifidobacteria.^12^ Bifidobacteria and *Lactobacillus* improve gut barrier function and host immunity and reduce the overgrowth of pathogenic bacteria, such as Clostridia.^14^

Enteral formula manufacturers are responding to this trend and cultural shift towards “real‐food” blended diets and developing formulas designed to address the feeding issues that children experience when receiving standard enteral formulas. Given the increasing requests for blended diets in our population and the paucity of available literature, we report on results collected from a national retrospective study to capture the clinical experience in children, across both acute and community settings, who had switched from a standard enteral formula to an “enteral formula with food‐derived food ingredients.”

## MATERIALS AND METHODS

This is a retrospective, multicenter study that monitored feed tolerance in children who have switched to Compleat Pediatric from Nestlé Health Science, a nutritionally complete enteral tube feed (containing 13.8% food‐derived ingredients in the form of rehydrated chicken, peas, green beans, and orange juice, providing 1 g fiber); Table 1 provides additional nutrition information. Ethical approval was granted by the Health Research Authority and Health and Care Research Wales 20/HRA/4828. The study was conducted from March 2021 to July 2021 across four National Health Service Trusts: three pediatric tertiary centers and one district general community hospital. Children were included if they had switched to an “enteral formula with food‐derived ingredients” because of previous feed tolerance issues related to retching, vomiting, flatulence, and/or abnormal stool consistency and frequency. Children had to have been receiving an “enteral formula with food‐derived ingredients” for at least 1 month, and the enteral formula must have accounted for at least 80% of their total energy requirements. All eligible children were aged between 1 and 17 years old.

**Table 1:** Nutrition composition of Compleat Pediatric, an enteral formula with food‐derived ingredients (nutritionally complete enteral formula)

Nutrition profile	Per 100 ml
Energy, kcal	117
Fat, g	5
Of which, medium‐chain triglycerides	0.7
Carbohydrate, g	14
Fiber, g	1
Protein, g	3.6
Sodium, mg	57
Osmolarity, mOsm/L	280

Abbreviation: mOsm/L, milliosmoles of solute per kilogram of water.

Data were collected by pediatric dietitians from dietetic records and inputted to a Microsoft form to capture anthropometric and gastrointestinal outcomes over a month‐long period when children were switched to an “enteral formula with food‐derived ingredients.” A link to the Microsoft forms was sent to each site by the clinical research company, Ixia Clinical Ltd. Once the Microsoft forms were completed by the dietitian, forms were automatically sent to Ixia Clinical Ltd. Data were compiled to represent all sites and downloaded into an Excel sheet for analysis performed by the principle investigator.

Clinical dietetic documentation on feeding tolerance was measured as either improved, no change, or worsened and on key markers of tolerance (retching, vomiting, flatulence, and stool consistency). Stool consistency and frequency were measured using a stool form scale, a standardized method of classifying stool form into a finite number of categories. The Bristol Stool Form Scale is an ordinal scale of stool types ranging from the hardest (type 1) to the softest (type 7). Data were also collected to capture any changes before and after the switch to the new enteral formula in relation to feed volume, calorie intake, and medication related to stool frequency and consistency.

### Statistical analysis

The primary outcome of interest was the change in feed tolerance. For each measurement period, the change in feed tolerance was assessed for each patient to identify any trends. Adverse events while receiving enteral formula with food‐derived ingredients were recorded. Anthropometric measures were recorded as median and interquartile range (IQR) for weight (kg) and height (cm). To examine the changes in weight (kg), energy intake (kcal), and feed volume (ml) during the study period, a paired *t*‐test was used to produce a *P*‐value and confidence interval. A *P*‐value <0.05 was deemed statistically significant. Statistical analysis was performed with SPSS software (version 23; IBM SPSS Statistics, Armonk, NY, USA).

## RESULTS

Forty‐three children were included in this national multicenter, retrospective study. Demographic, primary medical diagnosis, anthropometric, and feeding history data are provided in Table 2. The median age of children who had switched to an “enteral formula with food‐derived ingredients” was 6 years old (IQR, 4–8). The most frequently recorded primary diagnosis of children who had switched to the new enteral formula was related to neurological or neuro‐disability 20 of 43 children (47%). The median time children received an enteral formula before switching to the new enteral formula was 52 weeks: (IQR, 24–120). The primary mode of nutrition delivery was via a gastrostomy feeding tube: 34 of 43 patients (80%). A breakdown of the type of formula (amino acid, partially hydrolyzed, or whole protein) children were receiving before the switch to the new enteral formula is outlined in Table 2. One child, who was recovering from chemotherapy‐induced mucositis and receiving parenteral nutrition (PN), was challenged with a hydrolyzed formula, which resulted in diarrhea and the enteral formula was stopped. Subsequently, this child was challenged again 3 days later and switched directly from PN to the new enteral formula with no signs of feed intolerance.

**Table 2:** The demographic characteristics of all children who had switched to enteral tube formula with food ingredients (*n* = 43)

Characteristic	
Gender, *n* (%)	
Male	28 (65)
Female	15 (35)
Age, median (IQR), years	6 (4–8)
Weight, median (IQR), kg	18 (12–26)
Height, median (IQR), cm	100 (90–120)
Race/ethnicity, *n* (%)	
White or White British	32 (74)
Black or Black British African	5 (11)
Asian or Asian British Indian	6 (15)
Primary diagnosis, *n* (%)	
Neurological/neurodisability	20 (47)
Genetic syndrome	9 (21)
Ear, nose, and throat complication	3 (7)
Hematology/oncology	3 (7)
Disordered eating	4 (9)
Renal disease	1 (2)
Respiratory disease	3 (5)
Sepsis	1 (2)
Weeks on formula before switching to an enteral formula with real‐food ingredients, median (IQR)	52 (24–120)
Type of feed, *n* (%)	
Standard whole protein, 1 kcal/ml	10 (23)
Whole protein, high energy, 1.5–2.4 kcal/ml	13 (30)
Low energy, whole protein, 0.7 kcal/ml	2 (4)
Partially hydrolyzed, 1 kcal/ml	4 (9)
Partially hydrolyzed high energy, 1.5 kcal/ml	3 (7)
Amino acid	5 (11)
Blended diet	5 (11)
Parenteral nutrition	1 (2)
Feeding route, *n* (%)	
Gastrostomy	34 (80)
Gastrostomy with jejunal extension	4 (9)
Nasogastric tube	4 (9)
Parenteral nutrition	1 (2)
Feeding method, *n* (%)	
Gravity boluses	21 (48)
Continuous pump	18 (42)
Combination, intermittent	5 (10)

Abbreviation: IQR, interquartile range.

Sixteen children were on medication for constipation management before switching to the new enteral formula. After 1 month switching to the new formula, seven children reduced the quantity or frequency of medication, with one child stopping medication altogether. Parental reports of children who had gastrointestinal intolerances before switching to the new enteral formula recounted improvements in retching, flatulence, loose stools, and constipation after switching formulas (Table 3). One patient presented with vomiting and lethargy after switching to the new enteral formula. This child is now under the care of the local allergy team and has been diagnosed with food protein–induced enterocolitis syndrome. Prior to switching to the new enteral formula, this child was receiving a standard whole‐protein formula. Overall, the type of enteral formula (amino acid, partially hydrolyzed, or whole protein) the child was receiving prior to the switch had no influence on feed tolerance outcomes.

**Table 3:** Reported change in gastrointeninal symptoms after switching to an enteral formula with food ingredients

Gastrointestinal intolerance symptom	Reported number of patients with symptom, *N*	Post switch number of children who reported an improvement in symptoms, *n* (%)	Other, *n* (%)
Retching	18	17 (95)	1 (5) no change
Vomiting	13	11(85)	1 (5) reduction in stoma output 1 (5) vomiting worsened 1 (5) no change
Flatulence	8	6 (75)	2 (25) no change
Loose stools	11	10 (90)	1 (10) no change
Constipation	11	10 (90)	1 (10) no change

A comparative analysis reported weight gain in children who had switched to the new enteral formula after 1 month (*P* > 0.002) (Table 4). There was no significant difference in feed volume (*P* > 0.5) or total daily calorie intake (*P* > 0.7) after switching formulas (Table 4).

**Table 4:** Comparison before and after switching from a standard formula to an enteral formula with real‐food ingredients

	Before formula switch	One month after formula switch	*P*‐value (95% CI)
Weight, mean (SD), kg	19.5 (9)	20.1 (9)	0.002 (−1.0, −0.2)
Feed volume, mean (SD), ml	835 (383)	805 (376)	0.49 (−55, 113)
Feed energy, mean (SD), kcal/day	977 (497)	961 (462)	0.74 (−81, 113)

The Microsoft data forms had a section available for additional comments. A common theme captured from parents was that their child seemed more comfortable after switching to the new enteral formula. One parent reported that, prior to switching, they often had to stop the feed because of retching and very poor feeding tolerance, but this has now improved and feed volume has increased with no retching. Furthermore, another family reported that bowel habits improved so much since switching to the new enteral formula that their child was finally able to successfully toilet train. Overall, seven of 43 children (16%) children experienced positive changes in mood or behavior and were more happy and settled; four of 43 children (9%) saw changes in skin or hair; and two of 43 children (4%) saw a change in their schooling patterns, as these children were able to attend school and take part in activities. Finally, 12 of 43 (28%) children saw changes in feeding patterns, such as less time spent on feeding and more simple feeding regimens, such that their families felt confident to go on a holiday.

Ninety percent of dietitians who reported switching to the new enteral formula met the nutrition goals set prior to the switch, with 81% of dietitians reporting an improvement within 1 week of switching.

## DISCUSSION

Children who require nutrition support from feeding tubes routinely report feeding intolerances.^4^ Our national multicenter, retrospective study found that children who had switched to an “enteral formula with food‐derived ingredients” reported a significant improvement in gastrointestinal symptoms, including a reduction in retching, flatulence, and vomiting. Dietitians reported clinical improvements within the first week of switching to the new enteral formula that were sustained throughout the study period.

Our study reported improved feed tolerance in children who had complex gastrointestinal issues and had switched to an “enteral formula with food‐derived ingredients.” Our findings support those of Samela et al, who monitored the transition of 10 pediatric intestinal failure patients (>1 year of age) from an elemental formula to an “enteral formula with food‐derived ingredients.” They reported improved stooling patterns and concluded that a commercially available enteral formula with food‐derived ingredients is a cost effective and adequate means of providing nutrition to this patient population.^15^ Furthermore, our study supports findings by Coad et al, who reported positive clinical outcomes with the use of blended diets, including reduced gagging and retching in gastrostomy‐fed children with fundoplication.^7^

The mechanisms behind why blended diets and “enteral formula with food‐derived ingredients” work has been postulated to be the beneficial effect of fiber on the gut microbiota.^5^ A recent study reported that pediatric patients previously fed standard enteral formulas acquired a more diverse microbiome when switched to blended diets.^16^ Additionally, the increased viscosity of a blended diet means that digested chyme reaches the small intestine at a pace that stimulates a more regular hormonal response.^2^

Antibiotic treatment is strongly associated with diarrhea in patients receiving EN and is linked to intestinal dysbiosis, which leads to an increased risk of pathogen overgrowth and an altered metabolism of macronutrients, which induces osmotic diarrhea and the malabsorption of essential nutrients.^17^ For this reason, children admitted to the hospital are the most in need of a high‐fiber nutritionally complete formula to minimize intestinal dysbiosis from the barrage of intravenous antibiotics often administered in acute settings.

However, blended diets may not be suitable for intensive care or other acute clinical settings because of the perceived risk of microbial contamination and the variability in micronutrients and electrolytes.^8^ Therefore, having an alternative, such as a complete “enteral formula with food‐derived ingredients,” may serve as a compromise to a blended diet, bridging the gap between a full blended diet and a standard enteral formula, thus facilitating relationships and engagement between parents and healthcare professionals. However, as Chandrasekar et al correctly point out, there is limited evidence that blended diets can significantly reduce gastrointestinal symptoms associated with tube feeding and improve aspects of quality of life. More research is needed to evaluate whether blended diets and “enteral tube feed containing food‐derived ingredients” support growth in children and to explore potential complications.^8^ Of note, one child in this study discontinued the new formula because of an undiagnosed allergy‐related disorder and, therefore, it is advisable that any children who have not been exposed to whole food since being exclusively tube fed should be carefully monitored and may require further input from the allergy team.

The limitations of this study include its small sample size (therefore, results are ungeneralizable to gender and ethnic groups), short trial period, and retrospective design. Rather than stating causation, we can only allude to a potential association of an “enteral formula with food‐derived ingredients” and improved gastrointestinal symptoms. However, a strength of the study was its national, multicenter design and that data gathering was from a range of dietitians from different specialties and clinical settings.

Given the growing interest among caregivers to trial blended diets and “enteral formulas with food‐derived ingredients,” we urge that the healthcare community better understand this practice. We have observed the beneficial outcomes of switching to this new formula within a wide range of medically complex children. Our data should motivate healthcare professionals to engage and embrace this cultural shift, implementing more research to better evaluate the clinical impact and mechanisms of action of blended diets and “enteral formulas with food‐derived ingredients.”

## CONFLICT OF INTEREST

None declared

## FINANCIAL DISCLOSURE

Graeme O'Connor, Marie Watson, Martha Van Der Linde, Rita Shergill Bonner, and Julia Hopkins received payment per participant recruited from Nestlé Health Science UK during the conduct of the study. Sharan Saduera is a medical affairs dietitian and is employed by Nestlé Health Science UK.

## AUTHOR CONTRIBUTIONS

Graeme O'Connor and Sharan Saduera contributed to the conception and design of the research and drafted the manuscript. Graeme O'Connor, Marie Watson, Martha Van Der Linde, Rita Shergill Bonner, and Julia Hopkins contributed to acquisition and data collection, revised the manuscript, agree to be fully accountable for ensuring the integrity and accuracy of the work, and read and approved the final manuscript.
